# The Age-AST-D Dimer (AAD) Regression Model Predicts Severe COVID-19 Disease

**DOI:** 10.1155/2021/6658270

**Published:** 2021-03-23

**Authors:** Fátima Higuera-de-la-Tijera, Alfredo Servín-Caamaño, Daniel Reyes-Herrera, Argelia Flores-López, Enrique J. A. Robiou-Vivero, Felipe Martínez-Rivera, Victor Galindo-Hernández, Victor H. Rosales-Salyano, Catalina Casillas-Suárez, Oscar Chapa-Azuela, Alfonso Chávez-Morales, Billy Jiménez-Bobadilla, María L. Hernández-Medel, Benjamín Orozco-Zúñiga, Jed R. Zacarías-Ezzat, Santiago Camacho, José L. Pérez-Hernández

**Affiliations:** ^1^Multidisciplinary Team for the Attention and Care of Patients with COVID-19, Hospital General de México ¨Dr. Eduardo Liceaga¨, Mexico City, Mexico; ^2^Gastroenterology and Hepatology Department, Hospital General de México ¨Dr. Eduardo Liceaga¨, Mexico City, Mexico; ^3^Internal Medicine Department, Hospital General de México ¨Dr. Eduardo Liceaga¨, Mexico City, Mexico; ^4^Pneumology Department, Hospital General de México ¨Dr. Eduardo Liceaga¨, Mexico City, Mexico; ^5^General Surgery Department, Hospital General de México ¨Dr. Eduardo Liceaga¨, Mexico City, Mexico; ^6^Intensive Care Unit, Hospital General de México ¨Dr. Eduardo Liceaga¨, Mexico City, Mexico; ^7^Colorectal Surgery Department, Hospital General de México ¨Dr. Eduardo Liceaga¨, Mexico City, Mexico; ^8^Infectious Disease Department, Hospital General de México ¨Dr. Eduardo Liceaga¨, Mexico City, Mexico; ^9^Ginecology Department, Hospital General de México ¨Dr. Eduardo Liceaga¨, Mexico City, Mexico

## Abstract

**Aim:**

Coronavirus disease (COVID-19) ranges from mild clinical phenotypes to life-threatening conditions like severe acute respiratory syndrome (SARS). It has been suggested that early liver injury in these patients could be a risk factor for poor outcome. We aimed to identify early biochemical predictive factors related to severe disease development with intensive care requirements in patients with COVID-19.

**Methods:**

Data from COVID-19 patients were collected at admission time to our hospital. Differential biochemical factors were identified between seriously ill patients requiring intensive care unit (ICU) admission (ICU patients) versus stable patients without the need for ICU admission (non-ICU patients). Multiple linear regression was applied, then a predictive model of severity called *Age-AST-D dimer* (AAD) was constructed (*n* = 166) and validated (*n* = 170).

**Results:**

Derivation cohort: from 166 patients included, there were 27 (16.3%) ICU patients that showed higher levels of liver injury markers (*P* < 0.01) compared with non-ICU patients: alanine aminotrasnferase (ALT) 225.4 ± 341.2 vs. 41.3 ± 41.1, aspartate aminotransferase (AST) 325.3 ± 382.4 vs. 52.8 ± 47.1, lactic dehydrogenase (LDH) 764.6 ± 401.9 vs. 461.0 ± 185.6, D-dimer (DD) 7765 ± 9109 vs. 1871 ± 4146, and age 58.6 ± 12.7 vs. 49.1 ± 12.8. With these finding, a model called Age-AST-DD (AAD), with a cut-point of <2.75 (sensitivity = 0.797 and specificity = 0.391, *c* − statistic = 0.74; 95%IC: 0.62-0.86, *P* < 0.001), to predict the risk of need admission to ICU (OR = 5.8; 95% CI: 2.2-15.4, *P* = 0.001), was constructed. Validation cohort: in 170 different patients, the AAD model < 2.75 (*c* − statistic = 0.80 (95% CI: 0.70-0.91, *P* < 0.001) adequately predicted the risk (OR = 8.8, 95% CI: 3.4-22.6, *P* < 0.001) to be admitted in the ICU (27 patients, 15.95%).

**Conclusions:**

The elevation of AST (a possible marker of early liver injury) along with DD and age efficiently predict early (at admission time) probability of ICU admission during the clinical course of COVID-19. The AAD model can improve the comprehensive management of COVID-19 patients, and it could be useful as a triage tool to early classify patients with a high risk of developing a severe clinical course of the disease.

## 1. Introduction

The entire healthcare system's collapse is a serious public concern worldwide due to the pandemic caused by the severe acute respiratory syndrome coronavirus-2 (SARS-CoV-2) infection. In the United States (US), the coronavirus disease (COVID-19) has given way to a nationwide public health catastrophe. For the first time in US history, a disaster declaration has been put in place for all 50 states and most US territories [1]. Until 26 September 2020, there were 32,626,165 confirmed SARS-CoV-2 infection cases worldwide and 990,134 deaths, according to the Center for Systems Science and Engineering (CSSE) at Johns Hopkins University (JHU) [2]. Mexico is one of the countries with a higher frequency of deaths due to the COVID-19 pandemic, with more than 70,000 deaths, a tally surpassed only by the US, Brazil, and India [3]. In this catastrophic scenario, results essential to understand the main factors related to a worse prognosis in the Mexican population.

COVID-19 ranges from mild clinical phenotypes to life-threatening conditions like severe acute respiratory syndrome (SARS). Among COVID-19 patients, around 80% are present with a mild illness whose symptoms usually disappear within two weeks. However, around 20% of the patients may develop severe symptoms requiring hospitalization. The mortality rate for this group of patients is around 13.4%. Therefore, patient risk assessment, preferably in a quantitative, nonsubjective way, is essential for adequate patient management and medical resource allocation. The prognostic value of different variables is not yet fully understood [4].

Patients with COVID-19 often develop respiratory failure 8–14 days after symptom onset, with “silent hypoxemia” and a high respiratory rate [5, 6]. Other authors have described examples of patients going from being physiologically normal to decompensating just a few hours later [7]. Therefore, further to oxygen saturation, recognizing poor prognosis factors that appear earlier during the disease is the key to prioritizing medical care for these high-risk patients, thus achieving effective triage in saturated healthcare systems. Our study is aimed at identifying the early biochemical factors determined at admission time, which were independent of pulmonary parameters, related to the disease course's progression, and the development of severe illness conditioning need to admission to the intensive care unit (ICU).

## 2. Materials and Methods

### 2.1. Study Design and Data Collection

This was an observational cohort study. First, we prospectively identified 166 patients with COVID-19 due to SARS-CoV-2 infection admitted to our hospital from March to May 2020. Demographic, clinical, and biochemical data at admission time were obtained from the medical records of these patients. Independent variables of interest were sex, age, glucose, urea, creatinine, lactic dehydrogenase (LDH), aspartate aminotransferase (AST), alanine aminotransferase (ALT), alkaline phosphatase (AP), gamma-glutamyl transferase (GGT), ferritin, D-dimer (DD), total platelet count, mean platelet volume (VPM), hemoglobin (Hb), red cell distribution width (RDW), leukocytes, neutrophils, lymphocytes, brain natriuretic peptide (BNP), albumin, total proteins, direct bilirubin, indirect bilirubin, total cholesterol, triglycerides, sodium, potassium, chlorine, magnesium, phosphorus, calcium, fibrinogen, international normalized ratio (INR), C-reactive protein (CRP), creatine phosphokinase (CPK), creatine phosphokinase-myocardial band (CPK-MB), troponin I, and myoglobin. Our primary outcome was to identify disease biomarkers in patients with severe disease needing ICU admission (ICU patients) and compare them with those who remained stable and needed only standard care support through supplementary oxygen by mask (non-ICU patients). There was not a search for specific predictors already reported in the literature because this sampling was time-depending. The intensive care medical staff evaluated all cases that need transfer to the ICU; SARS development was the most important reason to transfer patients to ICU. All patients transferred to ICU were intubated and supported with mechanical ventilation. The decision to transfer a patient to ICU and to initiate mechanical ventilation was always taken by the medical staff of the ICU. Patients were treated according to a previously established algorithm based on international standard care dictated by the Infectious Diseases Society of America (IDSA) [8].

### 2.2. Predictive Model Construction

Differential factors were identified between ICU patients versus non-ICU patients; these variables were then used to create a model to early predict (at admission time) whose patients were at risk to need transfer to the ICU at any time during the follow-up. The derivation and validation of the prediction model were designed according to the TRIPOD guidelines [9]. The Institutional Review Board approved the protocol.

### 2.3. Inclusion Criteria

Patients admitted to the hospitalization area because of confirmed COVID-19 due to SARS-CoV-2 infection by nasopharyngeal and oropharyngeal swab positive tests using real-time reverse transcription-polymerase chain reaction (RRT-PCR) taken at admission time.

### 2.4. Exclusion Criteria

Patients with incomplete information on their medical records. This was a per-protocol analysis, so the intention-to-treat analysis was not done.

### 2.5. Derivation Cohort

We included consecutive patients admitted from March to May 2020.

### 2.6. Validation Cohort

We included consecutive patients admitted from June to August 2020.

### 2.7. Statistical Analysis

Continuous variables were expressed as mean ± standard deviation (SD). Categorical variables were expressed as frequencies and percents. Characteristics from ICU patients were compared with non-ICU patients. Differences between categorical variables were analyzed using the *χ*^2^ test or Fisher Exact test, whereas continuous variables were analyzed using two tails Student's *t*-test. A *P* ≤ 0.01 was considered significant.

To normalize the distribution of significant variables, we transformed it into their natural logarithm. The variables were ordered based on univariate significance by fitting a logistic regression model and added into the multivariate model using a forward selection procedure. Model selection was based on minimizing the Akaike information criterion and maximizing area underneath the receiver operator curve (AUROC) or concordance *c*-statistic, with priority given to the lowest Akaike information criterion. The final model named *Age-AST-DD* (AAD) was applied to both derivation and validation cohort, and AUROC analysis was performed to predict developing severe disease needing to be transferred to ICU. The model's diagnostic performance in derivation and validation cohorts was evaluated using sensitivity, 1-specificity, positive predictive value, negative predictive value, and diagnostic accuracy. All analyses were performed using IBM Corp. Released 2017. IBM SPSS Statistics for Windows, Version 25.0. Armonk, NY.

### 2.8. Sample Size

In a post hoc analysis (StatMate 2 for Windows), we found a power higher than 95% in the effect sizes of main variables (Age, AST, and DD), so we conclude that the sample size used to construct and then to validate de model was enough to get statistical validity.

## 3. Results

**Figure 1:**
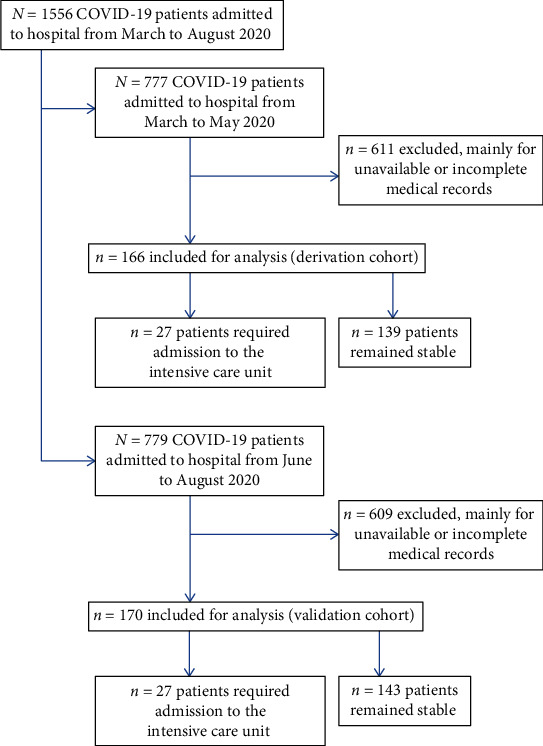
Enrolment of patients.

The enrolment of patients is summarized on the flowchart (see Figure 1).

### 3.1. Derivation Cohort

One hundred and sixty-six patients were included; from those, 114 (68.7%) were men. The mean age was 50.6 ± 13.3 years old. A total of 27 (16.3%) were ICU patients. In the comparative analysis between those ICU patients versus non-ICU patients, we found significant raises of ALT (225.4 ± 341.2 vs. 41.3 ± 41.1; *P* = 0.003), AST (325.3 ± 382.4 vs. 52.8 ± 47.1; *P* = 0.001), LDH (764.6 ± 401.9 vs. 461.0 ± 185.6; *P* = 0.001), DD (7765 ± 9109 vs. 1871 ± 4146; *P* = 0.003), and older age (58.6 ± 12.7 vs. 49.1 ± 12.8; *P* = 0.001). See Table 1.

**Figure 2:**
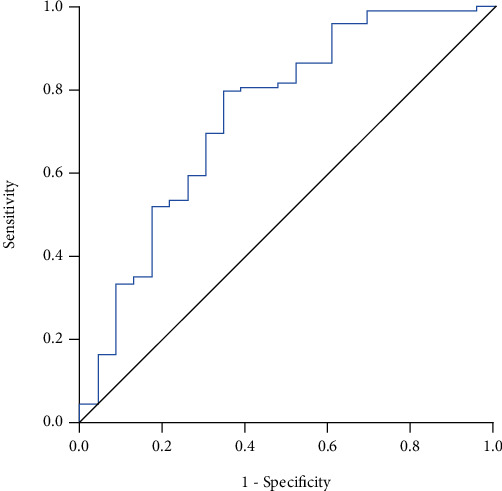
Derivation cohort (*n* = 166): AAD model to predict ICU admission. *c* − statistic = 0.74 (95% CI: 0.62-0.86; *P* < 0.0001).

The results of the linear regression are shown in Table 2, where model 3 was the one that best explained the need for ICU admission, with these variables was constructed the model called AAD, where [AAD = 3.896 + ln(age)x − 0.218 + ln(AST)x − 0.185 + ln(DD)x0.070], where a value ≤ 2.75 had sensitivity = 0.797 and 1 − specificity = 0.391, *c* − statistic = 0.74 (95% CI: 0.62-0.86; *P* < 0.0001), to predict the risk of developing severe disease and need to ICU admission (OR = 5.8, 95% CI: 2.2-15.4; *P* = 0.001). See Figure 2. The shrinkage factor for derivation sampling was 0.89.

### 3.2. Validation Cohort

**Figure 3:**
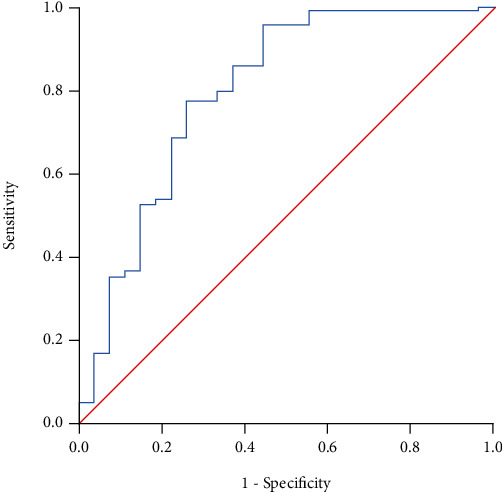
Validation cohort (*n* = 170): AAD model to predict ICU admission. *c* − statistic = 0.80 (95% CI: 0.70-0.91; *P* < 0.0001).

One hundred and seventy patients were included; from those, 116 (68.2%) were men. The mean age was 50.9 ± 12.8 years old. A total of 27 (15.9%) were ICU patients. The AAD value ≤ 2.75 in this cohort had sensitivity = 0.77 and 1 − specificity = 0.26, *c* − statistic = 0.80 (95% CI: 0.70-0.91; *P* < 0.0001), to predict the risk of requiring ICU admission (OR = 8.8, 95% CI: 3.4-22.6; *P* < 0.0001). See Figure 3. The shrinkage factor for validation sampling was 0.88.

## 4. Discussion

In this study, we develop a regression model using early biomarkers to predict the severity of COVID-19, assessing the need for admission to ICU. Cytokine storm, SARS, and systemic inflammation-related pathology characterize severe COVID-19 [10]. Liver injury is common and is associated with disease severity in patients infected by the other two significant coronavirus—SARS-CoV and the Middle East respiratory syndrome coronavirus [11–14]. Between 14.8% and 53% of COVID-19 patients had hepatocellular liver injury demonstrated by higher ALT or AST and slightly high bilirubin levels [15]. Moreover, liver injury frequency is higher in severe COVID-19 [16–19] and increases the mortality as high as 58 to 78% [20, 21].

Our study found that early liver injury, assessed by elevated aminotransferases, particularly AST, is a factor related to the worst progression in COVID-19 patients who require entering to ICU. Huang et al. [19] showed that AST elevation was observed in 8 (62%) of 13 patients in the ICU compared with 7 (25%) of 28 patients who did not require ICU admission. Wang et al. [22] also found that patients admitted to ICU had significantly higher ALT (35 vs. 23, *P* = 0.007) and AST (52 vs. 29, *P* < 0.001) levels. Our study results confirm the finding that liver injury is more prevalent in severe cases of COVID-19.

According to several studies, high values of CRP, ferritin, DD, procalcitonin, LDH, prothrombin time, activated partial thromboplastin time, amyloid serum protein A, CPK, GGT, urea, and creatinine are risk factors for severe disease, thromboembolic complications, myocardial damage, and worse prognosis [23–26]. In addition to aminotransferases, in our study, many of these factors were higher in ICU patients than in non-ICU patients, but the most important associated with severe disease were LDH and DD. The most severely ill patients usually present with coagulopathy, and disseminated intravascular coagulation- (DIC-) like massive intravascular clot formation is frequently seen in this group of patients [27, 28]. Therefore, as we found in our AAD predictive model, coagulation tests, specifically DD [29], may be considered useful to discriminate severe cases of COVID-19. Changes in hemostatic biomarkers represented by an increase in DD and fibrin/fibrinogen degradation products indicate the essence of coagulopathy is massive fibrin formation [28].

Liver injury in patients with COVID-19 might be due to viral infection in liver cells or due to other causes such as drug-induced liver injury (DILI) and systemic inflammation induced by cytokine storm or pneumonia-associated hypoxia [30]. A significant limitation of our study is that we were not able to correlate the biochemical findings at admission with liver biopsy in these patients; therefore, we are unable to determine if the serum alterations observed in liver function tests, particularly aminotransferases, are due to direct viral infection of the liver parenchyma. Another significant limitation is that the received therapy in these patients was heterogeneous regarding the date of starting, type of medication, and the dose of the medications, then we do not collect data from the received therapy of these patients; therefore, we cannot perform a subanalysis to try to identify potential DILI contributing to liver injury.

## 5. Conclusions

The elevation of AST (a possible marker of early liver injury) along with D-dimer and age efficiently early predict (at admission time) the probability of needing ICU admission during the clinical course of COVID-19. Our findings support using the AAD model to accurately determine those patients who would need to be transferred to ICU because of a severe clinical course of their disease. The AAD model can improve the comprehensive management of COVID-19 patients, and it could be useful as a triage tool to early classify patients with a high risk of developing a severe clinical course of the disease.

## Figures and Tables

**Table 1:** Comparison of admission characteristics between patients who developed SARS and required admission to ICU versus those with COVID-19 pneumonia without severity criteria.

Variable	Patients with SARS requiring ICU admission (*n* = 27)	Patients with COVID-19 pneumonia without severity criteria for ICU admission (*n* = 139)	*P* (^∗^ < 0.01)
Demographic and clinical characteristics			
Male/female gender, *n* (%)	20/7 (74.1/25.9)	94/45 (67.6/32.4)	0.51
Age, years old	58.6 ± 12.7	49.1 ± 12.8	0.001^∗^
Tobacco consumption, *n* (%)	7 (25.9)	26 (18.7)	0.43
Alcohol intake, *n* (%)	3 (11.1)	13 (9.3)	0.73
Diabetes, *n* (%)	8 (29.6)	48 (34.5)	0.82
Hypertension, *n* (%)	5 (18.5)	45 (32.4)	0.25
Weight			
Normal, *n* (%)	11 (40.7)	45 (32.4)	0.53
Obesity, *n* (%)	16 (59.3)	94 (67.6)	
COPD, *n* (%)	6 (22.2)	8 (5.7)	0.01
Cardiovascular disease, *n* (%)	3 (11.1)	10 (7.2)	0.45
Chronic liver disease, *n* (%)	4 (14.8)	13 (9.3)	0.30
Chronic rheumatic disease, *n* (%)	2 (7.4)	5 (3.6)	0.32
Dyslipidemia, *n* (%)	9 (33.3)	17 (12.2)	0.02
Chronic kidney disease, *n* (%)	3 (11.1)	6 (22.2)	0.12
Cancer, *n* (%)	1 (3.7)	14 (10.1)	0.46
AIDS, *n* (%)	1 (3.7)	1 (0.7)	0.30
Use of immunosuppressive medication different than steroids, *n* (%)	2 (7.4)	6 (4.3)	0.62
Chronic use of steroids			
No, *n* (%)	27 (100)	133 (95.7)	0.55
Low dose, *n* (%)	0 (0)	4 (2.9)	
High dose, *n* (%)	0 (0)	2 (1.4)	
Liver function tests			
Albumin, g/dL	3.27 ± 0.52	3.48 ± 0.50	0.09
Alanine aminotransferase, UI/L	225.4 ± 341.2	41.3 ± 41.1	0.003^∗^
Aspartate aminotransferase, UI/L	325.3 ± 382.4	52.8 ± 47.1	0.001^∗^
Alkaline phosphatase, UI/L	109.1 ± 74.8	96.8 ± 54.4	0.39
Gamma Glutamyl Transferase, UI/L	205.6 ± 360.4	125.4 ± 163.3	0.35
Direct bilirubin, mg/dL	0.8 ± 1.7	0.3 ± 0.3	0.23
Indirect bilirubin, mg/dL	0.8 ± 1.1	0.5 ± 0.3	0.31
Biochemical serum analysis			
Glucose, mg/dL	168.2 ± 95.0	149.8 ± 97.8	0.54
Urea, mg/dL	54.7 ± 37.0	42.1 ± 37.7	0.14
Creatinine, mg/dL	1.1 ± 0.7	0.9 ± 0.7	0.29
Cholesterol, mg/dL	102.9 ± 33.8	123.0 ± 27.0	0.03
Triglycerides, mg/dL	142.4 ± 45.8	145.7 ± 49.4	0.83
Total proteins, g/dL	6.5 ± 0.7	6.3 ± 1.0	0.60
Lactic dehydrogenase, UI/L	764.6 ± 401.9	461.0 ± 185.6	0.001^∗^
Serum electrolytes			
Sodium, mmol/L	128.8 ± ±26.8	135.8 ± 3.5	0.38
Potassium, mmol/L	4.2 ± 0.4	4.0 ± 0.5	0.19
Chlorine, mmol/L	102.2 ± 5.04	100.6 ± 4.35	0.25
Calcium, mg/dL	7.8 ± 0.47	8.0 ± 0.44	0.77
Phosphorus, mg/dL	3.2 ± 1.0	3.1 ± 0.8	0.75
Magnesium, mg/dL	2.3 ± 0.3	2.2 ± 0.4	0.27
Hematic cytometry			
Leukocytes, cells/mm^3^	10.3 ± 5.1	8.7 ± 4.5	0.23
Neutrophils, cells/mm^3^	8.9 ± 4.6	7.1 ± 4.2	0.09
Lymphocytes, cells/mm^3^	1.0 ± 0.4	1.0 ± 0.6	0.99
Hemoglobin, g/dL	14.7 ± 1.7	14.5 ± 2.3	0.82
Red cells wide distribution	14.8 ± 1.4	14.2 ± 1.4	0.15
Platelets, cells/mL	219.7 ± 73.1	226.4 ± 86.2	0.77
Mean platelet volume, fL	8.9 ± 0.9	8.4 ± 0.9	0.11
Coagulation tests and inflammatory profile			
International normalized ratio	1.1 ± 0.2	1.0 ± 0.3	0.63
Fibrinogen, mg/dL	640.7 ± 207.5	608.6 ± 168.9	0.54
D-dimer, ng/mL	7765 ± 9109	1871 ± 4146	0.003^∗^
Reactive C protein, mg/L	210.3 ± 157.4	142.7 ± 121.2	0.17
Ferritin, ng/mL	782 ± 518	786 ± 1011	0.98
Muscle enzymes			
Creatine phosphokinase, UI/L	169 ± 188	300 ± 462	0.36
Myoglobin, ng/mL	151 ± 151	110 ± 192	0.47
Cardiac enzymes and peptides			
Troponin I, ng/L	49.4 ± 136.7	26.1 ± 96.3	0.45
CPK-MB, ng/dL	34 ± 42	25 ± 17	0.29
Brain natriuretic peptide, pg/mL	56.9 ± 80.5	136.1 ± 342.2	0.49

AIDS: acquired immunodeficiency syndrome; COPD: chronic obstructive pulmonary disease; ICU: intensive care unit; SARS: severe acute respiratory distress syndrome.

**Table 2:** Multivariate linear regression models predictive of severe disease in patients with COVID-19 and requirement for ICU admission.

Model		Nonstandardized coefficients		Standardized coefficients	*P*	95% confidence interval for B		Colinearity statistics	
		*B*	Error deviation	Beta		Inferior limit	Superior limit	Tolerance	VIF
1	C	2.721	0.131		<0.001	2.462	2.980		
	AST	-0.229	0.033	-0.512	<0.001	-0.293	-0.164	1.000	1.000
2	C	3.161	0.198		<0.001	2.770	3.551		
	AST	-0.194	0.034	-0.435	<0.001	-0.261	-0.127	0.878	1.139
	DD	-0.081	0.028	-0.221	0.01	-0.135	-0.026	0.878	1.139
3	C	3.896	0.414		<0 .001	3.077	4.714		
	AST	-0.185	0.034	-0.413	<0.001	-0.252	-0.118	0.860	1.163
	DD	-0.070	0.028	-0.190	0.01	-0.125	-0.014	0.844	1.185
	Age	-0.218	0.108	-0.148	1.05	-0.433	-0.004	0.915	1.093

AST: aspartate aminotransferase; C: constant; DD: D-dimer; VIF: variance inflation factors. Resume of the model: (1) *R* = 0.512, *r*^2^ = 0.262, *r*^2^ adjusted = 0.256, standard error = 0.331. (2) *R* = 0.552, *r*^2^ = 0.305, *r*^2^ adjusted = 0.294, standard error = 0.322. (3) *R* = 0.570, *r*^2^ = 0.325, *r*^2^ adjusted = 0.310, standard error = 0.318. Durbin − Watson = 1.53.

## Data Availability

The datasets generated and analyzed in this study are not publicly available because of respect to and protect patient privacy but are available from the corresponding authors on reasonable request.
